# Diagnostic and Treatment Challenges in the Clinical Curing of MRSA Coxitis in a Tetraplegic Immunocompromised Patient: A Case Report and Literature Review

**DOI:** 10.3390/jcm14113887

**Published:** 2025-06-01

**Authors:** Egle Burbaite, Julija Lebedeva, Donatas Senkanec, Meida Rimkeviciene, Danguole Vaznaisiene

**Affiliations:** 1 Faculty of Medicine, Medical Academy, Lithuanian University of Health Sciences, LT-44307 Kaunas, Lithuania; egle.burbaite@stud.lsmu.lt (E.B.);; 2 Department of Orthopedics and Traumatology, Kaunas Hospital, Lithuanian University of Health Sciences, LT-47114 Kaunas, Lithuania; 3 Department of Radiology, Kaunas Hospital, Lithuanian University of Health Sciences, LT-47114 Kaunas, Lithuania; mr@rotorklubas.lt; 4 Department of Infectious Diseases, Lithuanian University of Health Sciences, Baltijos Str. 120, LT-47116 Kaunas, Lithuania

**Keywords:** hip joint, MRSA, HIV, tetraplegia, Girdlestone procedure, sepsis

## Abstract

**Background/Objective:** Coxitis is an inflammation of the hip joint, often resulting in pain and functional decline. It can be caused by various factors, including avascular necrosis, trauma, and infection. Methicillin-resistant *Staphylococcus aureus* (MRSA) poses a serious threat due to its resistance profile and destructive potential. To the best of our knowledge, there are limited studies on MRSA-induced purulent coxitis, specifically in patients with human immunodeficiency virus (HIV) and tetraplegia, making this case particularly valuable for expanding the understanding of this rare and complicated condition. The aim is to describe the clinical presentation, diagnostic workup, antimicrobial management, surgical intervention, and follow-up of a patient with an atypical hip joint infection. A brief literature review is also provided. **Case Report:** We report a case of suppurative coxitis caused by MRSA in a 38-year-old man with HIV disease and post-traumatic tetraplegia, which posed significant diagnostic and therapeutic challenges. The patient was diagnosed with MRSA bacteremia and suppurative coxitis after extensive work-up. Synovial fluid cultures were negative, likely due to previous antibiotic use. Targeted antimicrobial therapy was initiated based on blood culture and susceptibility testing. Surgical debridement and femoral head resection were performed. The patient showed progressive clinical and biochemical improvement with combined antimicrobial and surgical therapy. **Conclusions:** This case highlights the difficulty in diagnosing septic arthritis in patients with neurological disorders and immunosuppression, especially in the absence of classic symptoms. It emphasizes the importance of multidisciplinary care and early imaging in patients with persistent fever and unclear source of infection.

## 1. Introduction

Coxitis, or inflammation of the hip joint, most commonly affects the epiphyseal region and can lead to significant morbidity if left untreated. Clinically, it typically presents as localized pain in the groin or lower buttock, with possible radiation toward the knee. This pain is usually aggravated by physical activity and relieved with rest. The onset can be subtle, evolving gradually over months or even years, which often delays diagnosis and treatment. As the disease progresses, it can lead to the loss of hip function and overall mobility.

Inflammation of the hip joint is more prevalent in patients with other comorbidities such as rheumatoid arthritis and spondyloarthritis. The prevalence of coxitis depends on a person’s age, gender, and underlying medical conditions. Early diagnosis and timely appropriate treatment are crucial in protecting the joint from damage and maintaining its function [1,2].

The etiology of hip arthritis is diverse. It can be caused by mechanical, inflammatory, metabolic, or infectious origins. The most common causes include avascular necrosis, trauma, septic arthritis, and systemic conditions such as rheumatoid arthritis. Less commonly, metabolic bone disorders like Paget’s disease can contribute to degenerative changes. Also, certain anatomical abnormalities—such as developmental dysplasia of the hip and slipped capital femoral epiphysis—can contribute to early osteoarthritis in patients due to abnormal joint mechanics.

Infectious coxitis, though relatively rare, represents a serious diagnostic and therapeutic challenge. Obtaining laboratory specimens is often the next step in the evaluation of a patient presenting with possible musculoskeletal infection, after taking their history and performing a physical examination. This typically includes an erythrocyte sedimentation rate (ESR), a C-reactive protein (CRP), blood and wound cultures. Gram-stain, however, has a low sensitivity even if it is a bacterial infection. Synovial fluid cytological analysis is a standard for infection. This is not a binary finding. Some high cell counts may be due to inflammatory or Lyme arthritis, whereas others with lower cell counts may be caused by bacterial infections [3]. Serological markers are valuable for recognizing the severity of the infection, but more often fail to reflect the progress of the infection because of the immune system, characteristics of the pathogen, or unseen factors such as patients coming to the hospital after having antimicrobial treatment [4]. Among bacterial causes, *Staphylococcus aureus* is the most frequently implicated pathogen, with methicillin-resistant *Staphylococcus aureus* (MRSA) posing particular concern due to its resistance profile and potential for aggressive joint destruction. Reports of MRSA-induced purulent hip joint infection are limited, and the condition is especially rare in patients with complex comorbidities [5,6].

This case is clinically significant because, in addition to the rarity of MRSA coxitis and sepsis, the patient presented with severe comorbidities—namely HIV infection and post-traumatic tetraplegia following a gunshot injury. Importantly, the typical symptoms of coxitis, such as localized hip pain and a limited range of motion, were absent in this case due to the patient’s neurological deficits. The lack of classical symptomatology significantly complicated the identification of the infection source and delayed diagnosis, emphasizing the importance of clinical vigilance in atypical presentations.

The aim of this publication is to report this rare and diagnostically challenging case, accompanied by a brief review of the literature, in order to contribute to the limited understanding of MRSA-related coxitis in immunocompromised and neurologically impaired patients.

## 2. Case Report

A 38-year-old male presented with fever of up to 39 °C accompanied by chills, general weakness, profuse sweating, and a wound in the region of the left elbow joint. According to the patient, symptoms began approximately three weeks prior (around 20 November), when he developed febrile fever, with chills reaching 39.9 °C. He self-medicated with oral non-steroidal anti-inflammatory drugs and consulted a general practitioner, where a urinary tract infection was suspected and empirical antibacterial therapy was prescribed, though the specific regimen is unknown. Despite treatment, the condition did not improve, and febrile episodes persisted. The patient continued taking antipyretics, but without significant relief. Around the same time, a wound developed near the left elbow joint, which he managed with once-daily dressings using povidone–iodine solution. As his condition failed to improve, he contacted emergency medical services and was transported to the Emergency Department.

Upon initial examination, the patient experienced chills and excessive sweating. He was conscious, communicative, and oriented, responding to questions appropriately. The skin and visible mucous membranes appeared pale. The tongue was dry and coated. The pharynx and tonsils appeared normal. The hemodynamics were stable, with a regular heart rhythm and tachycardia with a heart rate of 106 beats per minute. Vesicular breath sounds were heard in the lungs without rales, and oxygen saturation was 99% on room air. The abdomen was soft on palpation, with no tenderness and normal bowel sounds. No peripheral edema was present. There were signs of tetraplegia. Importantly, the patient also had a purulent wound at the left elbow joint, which was moved by relying on the elbows, raising concerns about a potential source of systemic infection. In the area of the left elbow joint, a deep purulent wound measuring 3 × 4 cm in diameter was observed, with reddened wound edges. On the right elbow, there was a superficial abrasion. No wounds were found on the feet, although the skin was dry and scaly.

Initial laboratory results revealed leukocytes 7.94 × 10^9^/L, neutrophils 75.3%, hemoglobin 99 g/L, erythrocytes 3.34 ×10^12^/L, and platelets 439 × 10^9^/L. Biochemical analysis showed CRP 242.15 mg/L, creatinine 43 μmol/L, glucose 6.95 mmol/L, potassium 4.4 mmol/L, and sodium 128 mmol/L. Urinalysis showed 25 leukocytes per field and negative nitrites. Chest X-ray revealed no signs of pulmonary infiltrates. Due to the persistent fever of unclear origin, the patient was transferred to the Department of Infectious Diseases for further diagnostic evaluation and treatment.

Notably, the patient has a complex medical history. In 2013, he sustained a gunshot wound to the neck, resulting in a thoracic vertebra (Th5) injury and subsequent tetraplegia. He is also diagnosed with HIV and is on regular antiretroviral therapy, specifically Efavirenz 600 mg once daily and Abacavir/Lamivudine 600/300 mg once daily, with reported full adherence. The latest available outpatient laboratory results from November 2018 showed a CD4+ count of approximately 290 cells/μL; human immunodeficiency virus ribonucleic acid (HIV RNA) load was undetectable. In addition, from 17 October to 18 November 2018, the patient was hospitalized for unclassified enteritis, during which he developed symptoms of constipation, requiring enemas. He also reports an allergy to co-trimoxazole.

A thorough diagnostic evaluation was undertaken to identify the etiology and extent of the infection. Laboratory tests revealed markedly elevated C-reactive protein (CRP) levels ranging from 201 to 242.15 mg/L, consistent with a significant inflammatory response (Table 1). Creatinine levels were noted to fluctuate between 43 and 295.4 μmol/L, raising concerns about potential renal impairment secondary to sepsis. Hemoglobin levels were found to be decreased (74 to 99 g/L), indicative of anemia, often associated with chronic disease and severe infection. Leukocyte counts were from 7.9 × 10^9^/L to 3.8 × 10^9^/L over the course of hospitalization. Platelet counts ranged from 83 to 452 × 10^9^/L. Urinalysis showed the presence of leukocytes, erythrocytes, protein, and glucose, suggesting concurrent urinary tract infection or sepsis-related renal involvement.

Blood cultures returned positive for methicillin-resistant *Staphylococcus aureus* (MRSA), confirming the presence of a systemic bacterial infection (Table 2). The culture of the wound at the left elbow also yielded MRSA, indicating a common pathogen responsible for both the systemic and localized infections.

In the urine culture, 10^7^ CFU/mL of *Klebsiella pneumoniae* was isolated, which was moderately susceptible to amikacin, but susceptible to imipenem and meropenem and resistant to ciprofloxacin, gentamicin, ampicillin, piperacillin/tazobactam, cefuroxime, cefotaxime, nitrofurantoin, trimethoprim/sulfamethoxazole, and trimethoprim. Additionally, *Acinetobacter* spp. was susceptible to gentamicin, amikacin, ampicillin/sulbactam, imipenem, and meropenem, but resistant to ciprofloxacin and trimethoprim/sulfamethoxazole. The culture from the tongue revealed a high amount of *Candida albicans*.

The patient was empirically treated with ceftriaxone for 1 day and piperacillin/tazobactam for 2 days. The treatment was adjusted based on the culture results. The patient was treated with vancomycin, meropenem (for suspected *Klebsiella* and *Acinetobacter* urinary tract infection), and fluconazole. The vancomycin serum concentration ranged between 14.3 µmol/L and 17.3 μmol/L. However, despite the treatment, the fever persisted, and the source of the infection was investigated. Since the patient had undergone neck surgery after a gunshot injury and it was not clear whether there was an implant, additional tests were conducted to investigate a possible implant-related infection. The imaging studies, including X-ray and magnetic resonance imaging (MRI) of the neck, were normal. Echocardiography was performed, with no evidence of endocarditis. X-ray and ultrasound examination of the left elbow were performed, with no fluid or destruction detected.

During a detailed repeat physical examination, crepitus was noted in the right hip area. Since the patient was tetraplegic, he did not feel any pain. X-ray of the hip demonstrated joint effusion and significant soft tissue swelling, strongly suggestive of a purulent process within the hip joint. These findings supported the clinical suspicion of purulent coxitis differentiating from avascular necrosis. A computed tomography (CT) scan of the pelvis was performed, describing six fluid accumulations in the right hip joint and surrounding muscles. An orthopedic surgeon consultation was conducted, and the right hip joint was aspirated, yielding hemorrhagic (non-purulent) synovial fluid. No bacterial growth was observed in the synovial fluid, possibly due to prior antimicrobial treatment, and cytology showed numerous neutrophilic granulocytes (inflammatory changes) in the joint aspirate.

The patient was diagnosed with MRSA-induced purulent coxitis. Given the severity of his presentation, the patient underwent surgery. Resection of the femoral neck (Girdlestone procedure), removal of necrotic tissues and sequestra, debridement, and irrigation with antiseptic solutions were performed during surgery. Combined antibacterial therapy with vancomycin and rifampicin was prescribed for treatment. The possibility of remaining chronic osteomyelitis could not be ruled out. Subsequently, the treatment was changed to rifampicin 450 mg twice daily and tetracycline 100 mg twice daily (30 days) as a combination therapy. An overview of the antimicrobial treatment regimen is presented in Table 3.

**Figure 1:**
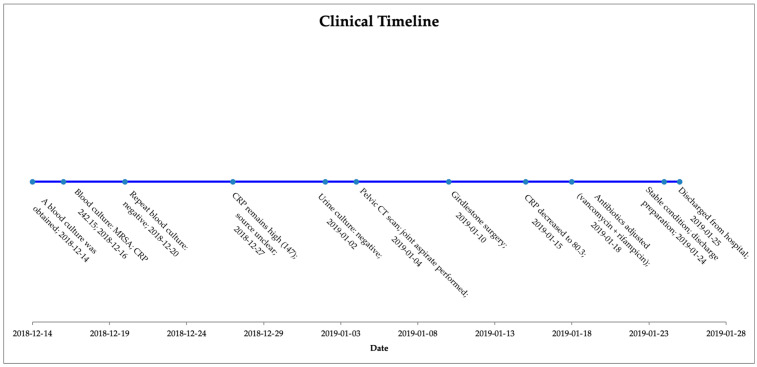
Clinical timeline summarizing key diagnostic findings, treatment interventions, and patient progress.

Despite the severe nature of his infection, the patient responded well to the antibiotic therapy and surgery. His fever subsided, allowing for a reduction in inflammatory markers such as CRP. Renal function stabilized with supportive care, and hemoglobin levels began to recover with blood transfusion before and after the surgery. Upon repeating the blood cultures, no growth was observed. The purulent wound at the left elbow also showed signs of improvement with local wound care and systemic antibacterial therapy. In order to more easily illustrate the progression of diagnostic tests, treatment decisions, and clinical response, a general timeline of the patient’s clinical course is presented below (Figure 1).

## 3. Discussion

Coxitis in immunocompromised patients is an understudied disease. Only a few articles study its prevalence, etiological diagnosis, and treatment. The medical management of infection should be focused on the adequate and timely drainage of the infected synovial fluid; administration of appropriate antibiotic(s); and debridement of any associated osteomyelitis or soft tissue infection with immobilization of the joint to control pain [7].

Coxitis usually presents with decreased hip mobility and discomfort, which is usually worsened by rotational movements. As the disease progresses, gait may be impaired and strides may become shortened. Hip anteflexion and retroflection among those patients are limited, and therefore their steps are short and slow. Movement restrictions are also found in adduction and abduction. Septic arthritis is usually characterized by obvious joint tenderness, a limited range of motion, and signs of inflammation. However, these classic features may be absent in individuals with neurological disorders or immunosuppression. The presentation is typically more subtle in those with periprosthetic joint infections, small joint infections, atypical infections (e.g., fungal, Lyme disease, tuberculosis), immunosuppression, or paralysis. An overlying skin infection can be the source of the entry point of the intra-articular infection. Septic arthritis should be considered in adults presenting with acute monoarticular arthritis. The timely identification and treatment of infectious diseases is very important, as prolonged inflammation can cause irreversible damage and increase the risk of systemic diseases. Subcartilaginous bone loss, cartilage destruction, and permanent joint dysfunction can occur if appropriate antibiotic therapy is not initiated within 24 to 48 h of onset. The reported incidence of septic arthritis is four to twenty-nine cases per 100,000 people a year, and risk increases with age, use of immunosuppressive medications, and lower socioeconomic status [5].

For this patient, the clinical diagnosis was initially complicated by tetraplegia and the absence of pain sensation. Differentiating avascular necrosis from septic arthritis involves a thorough clinical evaluation and imaging studies. Avascular necrosis typically presents with a gradual onset of pain and a limited range of motion in the affected joint, often following risk factors such as corticosteroid use or trauma. In contrast, septic arthritis usually manifests with acute onset of severe pain, swelling, and fever, indicating an infectious process. Laboratory tests, such as joint aspiration and culture, are crucial in identifying the presence of infection in septic arthritis, while MRI or X-rays may reveal changes indicative of avascular necrosis [8,9,10].

The pathogenesis of septic arthritis involves the bacterial invasion of the synovial membrane, which leads to an inflammatory process producing the characteristic purulent synovial fluid observed with arthrocentesis. Septic arthritis most commonly occurs due to hematogenous seeding secondary to bacteremia; other causes include penetrating trauma and corticosteroid joint injections.

The diagnosis of septic arthritis is established with the arthrocentesis of the affected joint. Cytosis (WBC of >50,000/μL) and polymorphonuclear cells of greater than 90% increase the likelihood of septic arthritis. Diagnosis and etiology are confirmed with Gram-stain and the culture of the joint fluid. Risk factors for septic arthritis include age older than 60 years, recent bacteremia, degenerative arthritis, rheumatoid arthritis, metabolic syndrome, immunocompromised state, joint endoprostheses, skin infection, and a history of sexually transmitted diseases.

Empirical antibacterial therapy should be directed primarily at the most common Gram-positive pathogens, and coverage should be expanded based on individual risk factors, including the effects of immunodeficiency or a potential Gram-negative source. Gram-negative coverage should be considered for patients with other risk factors, such as older age, immunosuppression, or bacteremia from a urinary or gastrointestinal source. Treatment should be individualized according to clinical responses and microbiology results. Septic arthritis caused by methicillin-resistant *S. aureus* requires drainage or debridement and three to four weeks of antibiotics. Parenteral options include intravenous vancomycin and daptomycin. Oral options include trimethoprim/sulfamethoxazole with rifampin, linezolid, and clindamycin, but there is no specific guidance regarding the duration of intravenous therapy before the initiation of oral therapy [7,9].

Rifampicin has been recognized as an effective treatment option for native joint infections, particularly when caused by *Staphylococcus aureus*, including MRSA. Studies have shown that rifampicin, when used in combination with other antibiotics, can enhance bioavailability and penetrate into biofilms, thereby improving therapeutic outcomes in septic arthritis. Furthermore, its unique mechanism of action, which inhibits bacterial RNA synthesis, makes rifampicin particularly valuable in treating chronic infections associated with prosthetic devices. However, the emergence of resistance and potential drug interactions necessitates careful monitoring and consideration of the patient’s overall antibiotic regimen. Additionally, the role of rifampicin in the management of native joint infections should be weighed against its pharmacokinetic properties and the necessity of combination therapy to optimize clinical outcomes and minimize the risk of relapse [11,12].

The literature review indicates that individuals with HIV have an increased risk of MRSA infections, though most of these infections are related to skin and soft tissue [13]. While there is a reported case of a paraplegic patient developing MRSA-induced necrotizing fasciitis, there are no specific reports in the literature regarding MRSA-induced coxitis in patients with HIV and tetraplegia [14].

HIV infection probably played a crucial role in the severity and persistence of the infection. A low CD4+ T-cell count weakens cellular immunity, which is essential to keep bacterial infections under control, particularly in deep tissue such as bone and joint spaces. In such patients, the early beginning of antiretroviral therapy (ART) alongside antimicrobial therapy is very important. Furthermore, observing for drug–drug interactions between ART and antibiotics (e.g., rifampicin) is necessary.

From a rehabilitation standpoint, patients with tetraplegia face challenges after hip resection. A loss of pelvic stability and changes in sitting balance can significantly worsen quality of life and increase the risk of pressure ulcers. Rehabilitation should focus on upgrading seating systems, incorporating custom-molded cushions, and engaging in targeted physiotherapy to strengthen trunk control. For patients like in this clinical case, prosthetic reconstruction is generally not practical, but functional positioning and passive support systems can help maintain comfort and prevent further musculoskeletal complications. Occupational therapy plays a critical role in modifying activities of daily living to accommodate biomechanics and prevent secondary complications [8,15].

Due to the rarity of MRSA-induced coxitis in immunocompromised patients, especially those with neurological impairments, further clinical observations and reports are needed. They are necessary for assessing the clinical suspicion of the disease, diagnostic characteristics, and the effectiveness of different antibacterial and surgical treatments. Sharing similar cases could help raise awareness, improve the recognition of atypical presentations, and support more timely diagnosis and treatment in the future.

Although this case provides valuable information about the course of a rare infectious pathology and its treatment in an immunocompromised patient, it is necessary to mention certain limitations of this work. Due to the hospital infrastructure relocation and health information system update work that was carried out this year, some of the patient’s previous imaging data (e.g., CT images) were not retained in the database. These images would have provided additional data about the extent of the infection, tissue damage, and helped to more accurately assess the clinical context. Nevertheless, based on the available clinical information, laboratory test results, and treatment course, this case is presented as a valuable contribution to the literature on the management of a rare infection in a patient with a complex profile. Preoperative and intraoperative images of the patient are not available either.

## 4. Conclusions

This case illustrates the difficulty in diagnosing septic arthritis in patients with neurological disorders and immunosuppression, especially in the absence of classic symptoms. It also demonstrates the interplay between systemic infection, immunocompromised status, and joint infection, emphasizing the need for vigilant monitoring and prompt treatment in similar clinical scenarios. Multidisciplinary collaboration is essential to optimize outcomes for patients with purulent coxitis, especially when underlying conditions complicate the clinical picture. Purulent coxitis, a rare but severe infection of the hip joint, presents significant challenges in immunocompromised patients, such as those with HIV. In this case, the patient’s immunocompromised status and MRSA infection contributed to the development of purulent coxitis. Early recognition and aggressive management are crucial to prevent joint destruction and systemic complications. This case underscores the importance of comprehensive care, including antibacterial therapy and surgical intervention, in managing such complex infections.

## Figures and Tables

**Table 1:** Blood results.

	14 December 2018	20 December 2018	27 December 2018	7 January 2019	10 January 2019 (1)	10 January 2019 (2)	15 January 2019	18 January 2019	24 January 2019	25 January 2019
Leucocytes (×10^9^/L)	7.94	7.9		5.5	3.8	6.7		5.3	4.9	
Hemoglobin (g/L)	99.0	97.0		86.0	79.0	82.0		74.0	85.0	
Platelets (×10^9^/L)	439.0	413.0		452.0	163.0	331.0		83.0	167.0	
Neutrophils (%)	75.3	70.0		58.3	59.7	77.6		67.4	65.9	
Potassium (mmol/L)	4.4	4.1	3.9	4.0		3.77	3.88		2.8	3.4
Sodium (mmol/L)	128.0									
Creatinine (µmol/L)	43.0	41.0	47.0			44.3	295.14	201.0	111.0	
AST (U/L)	79.0							25.0	19.0	
ALT (U/L)	89.0							17.0	12.0	
SPA (%)	68.0				65.0					
INR	1.2				1.2					
CRP (mg/L)	242.15	91.0	147.0	201.0		110.0	80.3		44.1	
ESR (mm/h)				>170					168	

Note. CRP = C-reactive protein; ESR = erythrocyte sedimentation rate; AST = aspartate aminotransferase; ALT = alanine aminotransferase; INR = international normalized ratio; and SPA = serum protein activity.

**Table 2:** Blood and wound culture results.

Antibacterial Agent	*Staphylococcus aureus*	Minimum Inhibitory Concentration (MIC) (µg/mL)
Penicillin	R	≥2
Oxacillin	R	≥4
Gentamicin	S	≤4
Tetracycline	S	≤0.5–1
Fusidic acid	S	0.03–1
Erythromycin	R	≥8
Clindamycin	R	≥2
Vancomycin	S	0.5–2
Trimethoprim/sulfamethoxazole	S	≤0.5/9.5
Rifampicin	S	≤0.5
Linezolid	S	0.5–2

Note. S = susceptible; R = resistant; and MIC = minimum inhibitory concentration.

**Table 3:** Antibacterial treatment timeline.

Date	Antibiotic	Details
14 December 2018	Ceftriaxone Piperacillin/tazobactam	1 g IV BID—empirical treatment for one day 4.5 g IV QID—empirical treatment for two days
16 December 2018	Vancomycin + Meropenem + Fluconazole	1 g IV BID—MRSA sepsis confirmed 1 g IV TID—due to suspected *Klebsiella* and *Acinetobacter* urinary tract infection 200 mg PO daily for 7 days—oral candidiasis
10 January 2019	Surgery—Resection Vancomycin	Girdlestone surgery (femoral head and neck resection; wound drainage)
18 January 2019	Vancomycin + Rifampicin	Added Rifampicin 450 mg PO BID—suspected osteomyelitis; vancomycin adjusted for renal function
25 January 2019	Rifampicin + Tetracycline	Tetracycline 100 mg PO BID; discharged with a total treatment duration of 6 weeks

Note. IV = intravenous; PO = per os (oral administration); BID = twice daily; TID = three times daily; and QID = four times daily.

## Data Availability

The dataset is available upon request from the authors.
